# Implementation of a psychosocial support package for people receiving treatment for multidrug-resistant tuberculosis in Nepal: A feasibility and acceptability study

**DOI:** 10.1371/journal.pone.0201163

**Published:** 2018-07-26

**Authors:** Ian F. Walker, Sudeepa Khanal, Joe P. Hicks, Bikash Lamichhane, Anil Thapa, Helen Elsey, Sushil C. Baral, James N. Newell

**Affiliations:** 1 Nuffield Centre for International Health and Development, University of Leeds, Leeds, United Kingdom; 2 Health Research and Social Development Forum (HERD), Kathmandu, Nepal; 3 National Tuberculosis Centre, Government of Nepal, Kathmandu, Nepal; Universita Politecnica delle Marche, ITALY

## Abstract

**Background and objectives:**

People receiving treatment for multidrug-resistant tuberculosis (MDR-TB) have high rates of depression. Psychosocial support in general, and treatments for depression in particular, form an important but neglected area of patient-centred care, and a key pillar in the global End TB strategy. We assessed the feasibility and acceptability of a psychosocial support package for people receiving treatment for MDR-TB in Nepal.

**Methods:**

This feasibility study used a mixed quantitative and qualitative approach. We implemented the intervention package in two National Tuberculosis Programme (NTP) MDR-TB treatment centres and 8 sub-centres. We screened patients monthly for depression and anxiety (cut-off ≥24 and ≥17 respectively on the Hopkins Symptom Checklist) and also for low social support (cut-off ≤3 on the Multidimensional Scale of Perceived Social Support). Those who screened positive on either screening tool received the Healthy Activity Program (HAP), which uses brief counselling based on behavioural activation theory. Other aspects of the psychosocial package were information/education materials and group interactions with other patients.

**Results:**

We screened 135 patients, of whom 12 (9%) received HAP counselling, 115 (85%) received information materials, 80 (59%) received an education session and 49 (36%) received at least one group session. Eight group sessions were conducted in total. All aspects of the intervention package were acceptable to patients, including the screening, information, group work and counselling. Patients particularly valued having someone to talk to about their concerns and worries. We were able to successfully train individuals with no experience of psychological counselling to deliver HAP.

**Conclusion:**

This psychosocial support package is acceptable to patients. The information materials we developed are feasible to deliver in the current NTP. However, the structured psychological counselling (HAP), is not feasible in the current NTP due to time constraints. This requires additional investment of counsellors in TB clinics.

## Introduction

Tuberculosis now kills more people globally than any other infectious disease [1]. Drug resistance is a major barrier to tuberculosis control. The number of people with multidrug-resistant tuberculosis (MDR-TB) is increasing, with an estimated 480,000 cases worldwide in 2014, the great majority occurring in low- and middle-income countries [2]. Successful treatment and management of MDR-TB is critical to controlling the epidemic. Experience suggests that MDR-TB patients under treatment have a high prevalence of depression, although evidence is limited [3–5]. Depression is likely to have a negative impact on patients’ livelihood and successful treatment completion. WHO’s global End TB Strategy [6] promotes ‘integrated, patient-centred care’ (which includes ‘patient support’ and ‘management of co-morbidities’) as one of the three pillars of TB treatment. However, its implementation is a challenge for National Tuberculosis Programmes (NTPs), particularly in low-income countries, where the focus is on the biomedical aspects of MDR-TB, and much less attention is paid to addressing the psychosocial needs of patients, including effectively treating depression [7, 8]. There are few studies testing psychosocial interventions for MDR-TB patients, despite the acknowledged importance of patient-centred care and psychosocial support [6, 9].

In Nepal, where in 2014/2015 there were 34,121 cases of TB, of whom 351 had drug resistant TB [10], the NTP recognises the significant current gap in psychosocial support for their MDR-TB patients [11, 12]. To address this, Nepal NTP is seeking to identify and develop contextually-relevant psychosocial interventions that can be delivered within routine NTP services. In response we assessed the acceptability and feasibility of a psychosocial support package delivered at health facilities within the existing NTP MDR-TB treatment programme, which includes screening and behavioural activation counselling, and aims to address the psychosocial needs of patients during MDR-TB treatment.

## Methods

### Study design

This feasibility study took a pragmatic approach, embedded within the existing health care delivery system and policy context. In preparatory work prior to this study, using interviews, focus groups, workshops and meetings with TB programme staff, we identified the potentially high rates of symptoms of mental disorders experienced by these patients. We found that low social support was a key issue for some patients, particularly women, and concluded that this needed addressing in a psychosocial intervention. The preparatory work also identified the lack of knowledge of MDR-TB symptoms and management amongst patients and their carers. We developed the intervention package and adapted the HAP with wide engagement with various stakeholders including the NTP, relevant Non-Governmental Organisations (NGOs), MDR-TB patients, their family members and TB health workers. This process has been described in detail elsewhere [13].

In the present study, we piloted this intervention package within the NTP service provision in Nepal. We evaluated the pilot to determine feasibility and acceptability of the intervention through a convergent mixed methods approach using both quantitative and qualitative data. The field work was undertaken by the Health Research and Social Development Forum (HERD), a Nepalese research NGO.

### Settings and participants

National MDR-TB treatment in Nepal follows an ambulatory approach which is provided through 14 MDR-TB treatment centres (“centres”) where patients are registered and attend monthly for follow-up, and 81 treatment sub-centres in 43 of 75 districts where patients receive daily supervised treatment. Very few patients are initially hospitalised. The feasibility study was implemented in two of these centres, one in Kathmandu (an NGO facility with 9 sub-centres), the other in Rupandehi (a government-owned facility with 8 sub-centres). These two centres were purposively selected based on high case load and expressed motivation of the health workers to deliver a psychosocial intervention. Four sub-centres were purposively selected per centre based on their proximity to the centre and motivation of health workers to deliver psychosocial support.

Potential participants in the pilot intervention were all those receiving treatment for MDR-TB (i.e. both those already on treatment and those newly diagnosed, without restriction) in the two centres and eight sub-centres between March 2015 and May 2016. All MDR-TB patients attending these clinics and receiving treatment were invited to participate in the study by the TB health worker when the researchers were also present. If the patient agreed in principle, they were then informed about the study using an information sheet, and informed written consent was obtained. Criteria for exclusion were: being without capacity to consent, unwilling to consent, aged <18 years old or being in the last 3 months of treatment. Patients who were interviewed for the evaluation were a sub-sample of those recruited to receive the pilot intervention.

### The piloted intervention

The psychosocial package was a stepped care model. This included education materials on MDR TB given to all patients and family members, screening for depression followed by counselling for those found to be depressed. Some patients were also able to participate in a support group if they wished. While some aspects of the intervention (described below) were offered to all patients (education and screening), other aspects were dependent on the screening scores (counselling) or on the patient’s willingness to participate (group support).

#### Information and educational materials

Education materials were used to give information on various aspects of MDR-TB and its treatment to all patients and family members (S1 Appendix). The materials included a flipbook (an A4 spiral bound book with pictures on the patient-facing sheet and explanatory text for the health worker on the back), and two information leaflets, one for patients and one for family members. While both the leaflets had information on MDR-TB, the family member leaflet had additional information on their supportive roles. These materials were designed to be used by the health workers in their routine consultations with patients.

#### Screening process

Screening for depression and anxiety was carried out using the 25 item Johns Hopkins Symptom Checklist (“HSCL”) [14], and screening for low social support using the Multidimensional Scale of Perceived Social Support (MSPSS) [15]. The screening was planned to take place during patients’ monthly follow-ups at the centre and to be conducted by the health workers. Patients who screened positive on at least one tool (anxiety ≥17 or depression ≥24 on HSCL [16]; or MSPSS≤3 [15]) were referred for counselling. Patients who screened negative across both tools were rescreened a month later. HSCL was selected as it has previously been translated and culturally adapted for use in Nepal for identification of depression and anxiety among internally displaced people, has been shown to have moderate validity [16], and was recommended by TPO Nepal, a Nepalese mental health NGO. There was no validated tool to capture the perceived need for social support in the needs assessment phase of the study [13]; the study team judged MSPSS was the most appropriate tool for the context. MSPSS was translated from English to Nepali by the research team and verified by back translation.

#### Brief counselling

Counselling was provided to those found to be depressed using the Healthy Activity Program (HAP) approach. HAP is individual counselling based on behavioural activation (BA) psychological therapy, developed in India for treating depression within primary care using lay counsellors [17]. The core principle of BA is the use of activity scheduling (planned timetables) to address inactivity and problems that the person avoids. The recipient of BA identifies activities to undertake that they are likely to find enjoyable in line with their goals and values. Behavioural activation is equivalent in effectiveness to cognitive behavioural therapy for depression, but significantly cheaper [18]. HAP was selected as it is an intervention with promising evidence from neighbouring India [19] and was already being tested in a few districts of Nepal in primary health care settings. We worked with the local mental health NGO who were testing HAP [20], to adapt HAP for MDR-TB-related context and issues: the principles and process remained unchanged with the exception of removing detailed recording of the counselling sessions to reduce the already heavy burden of documentation within the study. The HAP counsellor used this BA approach, as well as the activation (or re-activation) of social networks and a positive problem-solving approach (all aspects of the original HAP intervention from India), to help the patient identify the links between what they did and how they feel, and then used this information to plan specific activities to begin to improve their mood. Monitoring the progress of treating the patient’s depression through HAP was done using PHQ-9 [21], which has been validated in primary care populations in Nepal [22]. It was administered before the first session and thereafter before every HAP session. If the first PHQ-9 screen score was below 10, patients did not receive HAP but were returned to monthly screening. Patients whose first PHQ-9 score was over 19 (indicating severe depression) or who expressed suicidal intent were referred to specialist psychiatric or medical services (as available at each study site). For patients who started HAP, sessions were continued until the PHQ-9 score reduced to less than 10, indicating restoration of the patient’s mental wellbeing or for up to 8 sessions (lasting for 30 to 60 minutes each). If the PHQ score was still 10 or above after 8 sessions then referral to psychiatric services was made.

HAP counsellors are typically lay people with at least high school education who receive specific training in delivering HAP from experienced counsellors. The initial plan was for the TB health workers to conduct the counselling, however, early discussions with participating centres made it clear that this would not be feasible. In light of this two additional staff from HERD were trained to deliver the counselling in the centres. Both counsellors had public health backgrounds: one had extensive experience of TB programme work, and the other had a physiotherapy bachelors degree, but neither had previous formal counselling experience. Although most patients in Kathmandu speak and understand Nepali, in Rupandehi a significant minority do not, so the counsellor there was chosen for a familiarity with several local languages. Different tools were used for monthly screening (HSCL and MSPSS) and HAP monitoring (PHQ-9) as we wanted to identify anxiety as well as depression in the monthly screening, which the HSCL tool does. The PHQ-9 was the recommended and evidenced tool to monitor HAP and so was used for this purpose.

#### Group support

Interactions among patients in groups were used to increase patients’ understanding of MDR-TB and its treatment, reduce their negative emotions and improve their social support. Attendance at a group session was offered to all patients. A group of 8–15 people (mixed age and gender) with MDR-TB met regularly for up to an hour at the health facility, where they shared experiences and supported each other to face and overcome the challenges they were living with as a result of MDR-TB and its treatment. We planned a structured approach where the same group of patients would attend four consecutive sessions, covering important issues which would build trust and rapport between group members, increasing their ability to support one another. The group was facilitated by a counsellor, and often included a cured patient who shared experiences to provide motivation and instil hope in the group members. Group support has been shown to be beneficial in Peru where it has been a mainstay of MDR-TB care [23]. This aspect of the intervention package was developed in the second half of the study period after reviewing progress.

#### Training

Six days training was provided to MDR-TB health workers and counsellors by the mental health NGO, TPO. This consisted of a recap of MDR-TB modular training provided by the NTP, orientation to the information materials we developed for the study, basic skills of counselling including rapport building, using screening tools and delivering HAP. A further two days training was provided to the counsellors by the study team covering recording and process documentation of the research study.

### Evaluation of the intervention

We used both quantitative and qualitative data to assess feasibility of the intervention. These were combined using a convergent mixed-methods approach [24]. The objective of this evaluation was to determine the feasibility and acceptability of the intervention in the routine MDR-TB treatment service of the NTP.

#### Quantitative data

The quantitative data was collected from four sources between March 2015 and May 2016. The first was routine TB registers at the TB clinic. Data collected included: date diagnosed with MDR-TB, date started treatment, new or relapse case and sputum culture status. The second source was a data collection tool designed for this study, which collected self-reported socio-demographic data from the patient such as age, sex, education and occupation. The third source was the screening tools (HSCL, MSPSS and PHQ-9) which produced scores at each screening point for each patient. The fourth source was a researcher diary which collected process data from the counsellors and was used to identify such things as the percentage of patients who received different parts of the package and the time taken to deliver interventions. This measured fidelity to the planned intervention.

All quantitative data were manually input into a spreadsheet. Any outliers or gaps were checked with the counsellor and/or clinic registers. The whole dataset was then manually checked for consistency and completeness. Subsequently SPSS (version 20) was used to analyse the data and calculate descriptive statistics. As this was a feasibility study, no formal sample size estimation was carried out.

#### Qualitative data

A semi-structured format of the researcher diary with space for free-format text was provided to enable the counsellors to reflect on the implementation of the pilot intervention (S2 Appendix). The diary was completed by the counsellors initially on a daily basis, and later reduced to weekly, bi-weekly and then monthly reporting. The counsellors also wrote reflective notes to summarise their observations of delivering the intervention. After each HAP counselling session, the counsellors also made some brief notes on the content of the session to assess fidelity to the model.

Interviews were conducted with the counsellors, some of the MDR-TB patients who received HAP and TB health workers. Interview guides were developed for each group based on the information within the daily diary, informal conversation with the counsellors, aspects of the intervention that we wanted to assess and areas where there were information gaps (S3 Appendix). Two rounds of interviews were conducted with the counsellors, one on month 9 of the intervention and the other at the end of the intervention, to more thoroughly understand the implementation and the context at the MDR-TB centres. The number of interviews was fixed with counsellors and TB health workers as there was a limited number of these staff (all were interviewed). Interviews with patients were continued until the interviewers considered data saturation had been reached.

Qualitative data from interviews were translated from Nepali into English by experienced translators and transcribed to English as literally as possible. These transcripts, daily diaries, reflective notes and HAP session comments were uploaded into NVivo (version 11). In accordance with the framework approach in qualitative analysis [25], two researchers thematically coded two interview transcripts and from this an agreed thematic framework was developed. This was then checked independently by a third researcher. The framework was then used to code all subsequent transcripts and diaries. Any deviant data were identified; further themes were considered and agreed between the two researchers, and the framework adjusted accordingly.

#### Ethics

Ethics approval for the study was obtained from Nepal Health Research Council (Approval number: 130/2012) and University of Leeds Medical Research Ethics Committee (Approval number: HSLTM/11/026). Written informed consent was obtained from all research participants. Illiterate patients were informed verbally and confirmed consent using thumb prints. No incentives were provided to participants.

## Results

During the implementation period (March 2015-May 2016) 197 patients were registered in the two MDR-TB treatment centres. Of these, 135 patients were eligible, consented and were enrolled in the study. Reasons for patients not being enrolled included: non-attendance at the clinic, disruption to the project from the large earthquake in April 2015 and meeting the exclusion criteria for recruitment. Details of enrolled patients’ characteristics are shown in Table 1. Males outnumbered females (76% v 24%). The sample was generally quite young. A majority of the sample was married (67%). A majority lived with a spouse or family members (30% staying alone, 68% staying with family/spouse). More participants were recruited in Rupandehi than Kathmandu (61% v 39%). Only three participants (2%) reported not working before MDR-TB, compared with 94 (70%) after being diagnosed.

**Table 1:** Patient characteristics.

Characteristics		N = 135
Age		30 (23–43)
Sex	Male	102 (76%)
	Female	33 (24%)
Occupation	Not working before MDR diagnosis	3 (2%)
	Not working after MDR Diagnosis	94 (70%)
Family situation	Staying alone	41 (30%)
	With family/spouse	92 (68%)
	Others (hostel)	2 (2%)
Marital status	Not married	44 (33%)
	Married	88 (65%)
	Widowed	3 (2%)
Treatment centre	Kathmandu	52 (39%)
	Rupandehi	83 (61%)

Data are n (%) or median (interquartile range).

The data for qualitative analysis was obtained from 11 interviews (5 patients, 2 MDR-TB health workers and two counsellors interviewed twice), 93 reflective diary entries and three field visit reports.

**Fig 1:**
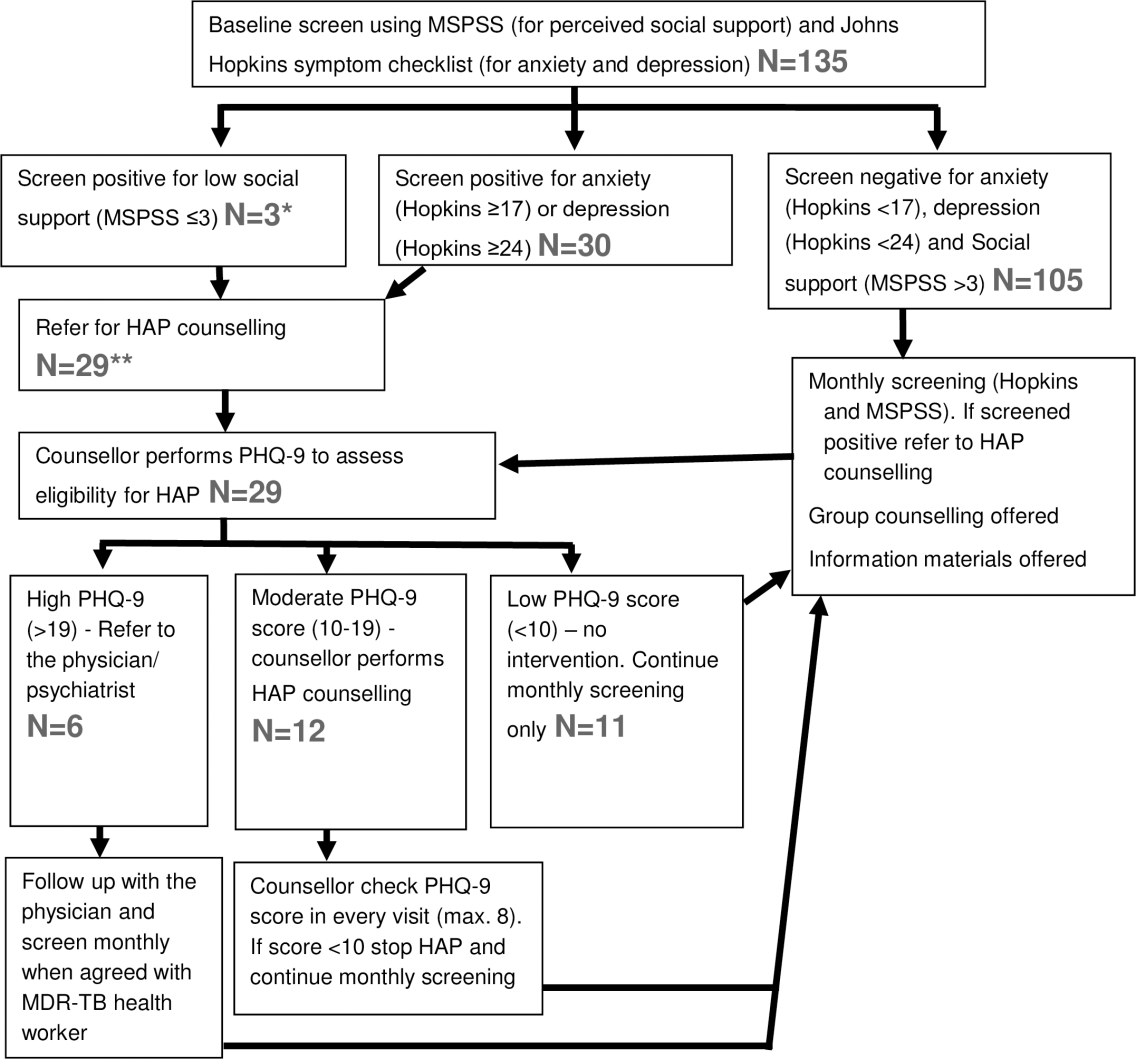
Flow diagram of the screening process in this study. * These 3 patients with MSPSS<3 also screened positive for depression or anxiety on the Hopkins tool. ** 1 patient with a high Hopkins score was (mistakenly) not referred to the counsellor and so not counted here.

During the study period the 135 patients were receiving treatment for a cumulative total of 943 months. We obtained monthly screening data for 693 of these months (73%). Missing data for monthly screening was due to patient non-attendance at the centres. Of the 135 patients screened with HSCL and MSPSS, 3 screened positive for low social support and 30 screened positive for depression and/or anxiety indicating the need for further counselling (see Fig 1). All three patients who had screened positive for low social support (MSPSS score) also screened positive for depression (HSCL). Therefore, a total of 30 patients were eligible for HAP counselling after screening. One patient who screened positive on HSCL was (mistakenly) not referred for HAP counselling: the remaining 29 were referred for counselling. Of the 29, six patients had a very high baseline score on PHQ-9 (>19) (administered as part of the HAP process) indicating severe depression. Instead of receiving HAP counselling they were referred to a MDR-TB health worker/physician/psychiatrist depending on availability at the health centre, due to their psychiatric needs. Of the 29 patients, 12 had a moderate PHQ-9 score (10–19) and were counselled using HAP. There were 11 patients who had a low PHQ-9 score (<10) and were not eligible for HAP counselling, and were therefore returned to routine monthly screening.

Of the 135 patients, 115 (85%) received a patient information leaflet, and 36 patients’ (27%) family member(s) received a family information leaflet. 80 (59%) patients received one flipbook session, which the counsellors reported took on average 15–20 minutes. Some of these sessions at Rupandehi were delivered by student nurses on placement at the clinic after training from the counsellor and TB health worker.

A total of eight group support sessions were delivered during the study with a total of 49 (36%) patients receiving at least one group session. Three groups (mean size 5 patients) were delivered in Kathmandu involving a total of 14 patients and 5 groups (mean size 7 patients) in Rupandehi involving 35 patients.

### Feasibility and acceptability of the psychosocial package

#### The screening process

Within the first month of implementation the MDR-TB health workers stated that they were unable to perform the role of initial screening of all MDR-TB patients due to the heavy patient flow and time constraints. This was in spite of the fact that the intervention was developed with their full engagement and they had agreed this role. With limited opening hours between 10am and 2pm and MDR-TB patients from the sub-centres presenting on just two days per month, health workers could not manage the screening of the patients. Therefore, the counsellors themselves undertook the screening process which they had enough time to do. For the last month of the study the MDR-TB health workers were asked to use a locally developed ultra-brief screening tool[22] using two questions similar to PHQ-2, but using Nepali idioms of psychological distress. They did this and reported having enough time to undertake this for every MDR-TB patient, indicating the use of two questions as an initial screen is feasible without additional resources in the current TB service.

“If there is a separate person [for screening], then it is good to go through HOPKINS and MSPSS, but if he [MDR-TB health worker] has to implement in the regular system … then the two questions will be better. It is short and doesn’t take too much time.” (Counsellor 2)

One of the benefits of this revised delivery method using the counsellors was there was minimal waiting time for the patient to be screened. The counsellors managed to perform over 70% of the planned monthly screening sessions, providing plenty of opportunity to identify patients with depression, indicating the feasibility of this approach. Counsellors were also positive about using HSCL and MSPSS (compared to just PHQ-9) for screening because it provided richer information for the HAP counselling session as they knew more about the patient’s state of mind and social support. They reported it took them 10–15 minutes to complete the screening process for each patient.

Counsellors reported that, due to cultural factors, one question in the HSCL tool (relating to sexual interest) was uncomfortable to ask and for patients to answer. Other questions in the tools were acceptable. Counsellors also reported that patients referred for HAP complained about the repetition of the questions in the two screening tools (HSCL and PHQ-9).

#### Information and educational materials

The convenience of giving the leaflet to patients led to the MDR-TB health workers providing one to a majority of patients. However, although the MDR-TB workers initially used the flipbook in their contact with new patients, this practice soon stopped. Although it only took 5–10 minutes to go through, this seemed to become a barrier to them using the flipbook. They stated that they gave the same messages but more briefly.

“It [the flipbook] is very difficult for us [to use for every patient]. It is practical in places that have a small number of patients. On the other hand, this facility has a large volume of patients here.” MDR-TB Health worker 2

“The things I used to give are also from the flip book. I finish up saying the things in two lines.”MDR-TB Health worker 2

All the information materials were in Nepali and for a majority of patents these could be understood; however, for a minority of patients at Rupandehi who did not speak Nepali, this presented a barrier. This was overcome by the counsellor translating the content into a local language.

Most of the patients found the information materials (particularly the pictures) helpful to understand MDR-TB and its management better, which reduced their mental stress (see quote from Counsellor 2 below). Some also stated that the intervention should be available in all MDR-TB centres.

*“In the beginning*, *I was interested to know what it [the leaflet] was*. *I felt like reading it and I read it too*. *When I found out that it was about my own [condition]*, *my ‘interest’ grew even more*.*”* Rupandehi Pt 3, Female

“If we screen some patients and if we counsel them they said that their results came good. They also said that, ‘Sir, we didn’t know about TB and we didn’t know with whom to share. Because of this, we were depressed but after you came, we could share with you everything, we knew everything regarding its transmission. And after sharing these things I felt relaxed…’” Counsellor 2

#### The HAP counselling

It took 30–45 minutes to deliver each HAP session. Due to the availability of the counsellor as an extra member of staff, the time patients spent waiting to receive HAP counselling was minimal.

Twelve of the 135 (9%) patients received the HAP counselling. The median number of HAP sessions delivered per patient was 2.5 (range 1–4). Only one patient brought a family member to the HAP counselling (for two sessions). With the counsellors also undertaking the screening, there was generally no waiting time between screening and delivery of the HAP sessions as they occurred consecutively. There was a single instance where two patients were identified to receive HAP on the same day resulting in a longer waiting time for one of them. However, a key limitation of delivering the HAP sessions was the lack of an appropriate, private space in which to conduct the counselling at the centre (as was the case with screening), which often created distractions for the patients and counsellors during counselling. The counsellors had to improvise, and either used rooms that were shared with other services or an outside space that was as private as possible.

A review of the counsellors’ reflections indicated the focus of the HAP sessions was on patient activities in line with the behavioural activation approach of HAP. Typical examples of activation included patients doing breathing exercises, intentionally talking with neighbours, starting to undertake manageable household chores, ensuring a good sleep routine and starting to work part-time in the family business. As the counsellors took a purely oral approach to activity planning without the use of paperwork, the counsellors reported that the patients with low literacy were able to engage in the counselling.

There was high attendance for HAP sessions. All patients appreciated the availability of someone with sufficient time in the centres to be able to share their problems and sorrows. This was particularly important for those who, due to the stigma of TB, were unable to talk openly about their condition with family and friends. In addition, all the patients interviewed also stated that they were willing to see our counsellors and follow the agreed activities of the HAP session.

“Before this, other people have come asking, but I never showed them my worries. And when counsellor asked us very nicely, giving us enough time … I shared all my problems with him … after talking with him I have improved a lot. I have felt very good after I met the counsellor … and I used to follow every little thing said by him.” Kathmandu Patient 2, Male

“What happens is we don’t share with other friends from outside about our disease. But he asks us about how our family is and what is going on. At least there is a person in the hospital who asks us about our family … and that’s a big thing for us” Kathmandu Patient 3, Male

Most of the patients identified that the behavioural activation approach using changes to daily activities was particularly beneficial.

“Before meeting the Counsellor, I used to think a lot … I used to spend time gazing a lot and continue walking unknowingly. Then the counsellor told me not to overthink and not to take “tension” … and used to teach me to do these things and told me if you will do these things then this [improvement] will happen. From then I didn’t take any kind of tension … after talking with him I have improved a lot” Kathmandu Pt 2, Male

“After the counselling, I tried to change myself. While I was in depression, I used to walk around thinking, ‘I do not care whatever happens’. Because of the counselling, I got the chance to change myself.” Rupandehi Pt 3, Female

“In my experience, one thing is that, they [patients] came out searching for him [counsellor] and no more psychological problems came to me … the problem has been reduced compared to last year. Not only the suicidal thoughts but also suicidal attempts had been done here in mine [my facility]” MDR-TB HW 1

#### Group support

It was not possible to retain the same members for subsequent group sessions as planned because patients attended the clinics on different days of the month and times of day. Therefore topic-based group sessions proved challenging. Instead, the counsellors facilitated participants to identify the challenges they were willing to discuss in a group setting on a session-by-session basis. Groups were limited to 30 minutes, based on the willingness of patients to stay that long. As the group could only start once all the invited patients had seen the MDR-TB health workers, the session start was often delayed, resulting in patients requesting shorter group sessions. Overall, patients were positive about group counselling; however, some thought it may not suit everyone as some people would not like to express differences in opinions and some might worry more after hearing others’ problems. In addition, many patients expressed how beneficial it was to hear the experiences of a cured patient in the group.

“Seeing the people who have completed their course of medication will motivate the people who are continuing their medicines. On seeing such people, they will realize that they too can survive if they take medicines. They get motivated and they go on completing their course” Rupandehi Patient 3, Female

“In the beginning, I used to go there, bring medicines and take them. There was no one like that [with MDR-TB] in the village, so I used to stress out thinking that I was the only one with that disease. When I saw all the other people here [the treatment centre], I realized that there were others like me who were suffering from it. I got courage thinking that others have also gone through it and they have survived.” Rupandehi Pt 5, Male

#### Other challenges and benefits

According to the intervention protocol, patients whose scores indicated anxiety or severe depression were supposed to be referred to a psychiatrist. No psychiatrists were available within the study health facilities so cases were notified to the MDR-TB health worker, who then followed up with a physician or psychiatrist at another facility.

The MDR-TB health workers expressed their views that the intervention was useful and a valuable part of the service that they were offering. While they had limited time to deliver the intervention they did provide support to facilitate the intervention process by arranging space for counselling and encouraging patients to engage in the components of the intervention.

“The things that we have been implementing with our patients [the intervention package] have been really good for the patients and we have had no defaulters at all. They now come to inform us immediately whenever they have a problem. They used to hesitate to come and talk to us before. We feel that these things [the intervention] have made the situation far better than before.” MDR-TB Health worker 1

#### Preliminary HAP outcomes

Our sample size was not powered to report effectiveness of HAP. That said, the 9 patients who completed HAP had a mean reduction in scores on both depression tools (7.9 points on HSCL-Depression and 2.7 points on PHQ-9). This is an encouraging signal of the possible impact of HAP on depressive symptoms in this setting, but no firm conclusions on effectiveness can be made. Minimal change occurred for the HSCL-Anxiety scores after HAP, compared with before HAP.

## Discussion

As far as we are aware this is the first published study to implement a BA counselling approach in the treatment of MDR-TB in a resource-constrained setting. This psychological intervention within a psychosocial support package shows promise as an intervention for people being treated for MDR-TB, particularly for those with depression. We have shown that the HAP counselling and the package as a whole is acceptable to patients. We have also demonstrated that HAP counselling is not feasible in the current NTP due to the time taken to deliver the counselling sessions. However, with additional resources of counsellors provided for TB clinics, who are aware of local languages and have a private space to use, then HAP counselling could be feasibly delivered as part of a package of psychosocial interventions. We have shown that this counsellor does not need to be someone with professional qualifications. The use of information materials and an ultra-brief screening tool for depression (2-item) is feasible to deliver in the current NTP. With additional dedicated human resources, screening, group support and HAP counselling can feasibly be delivered in centres by counsellors or health workers following appropriate training. The information materials developed during the study are the first resources focusing on MDR-TB in Nepal and are now available to be scaled up across the MDR-TB services.

Strengths of the study include the intervention being built on the findings of previous studies; an embedded approach within the existing TB service; design and development in collaboration with the NTP; an intervention with existing evidence and an established theoretical base; and contextualization to Nepal and tailoring for implementation in existing MDR-TB services. A key challenge was disruption in the delivery of the intervention for 3 months due to the large earthquake in April 2016 and other political unrest across the country which caused disruption in monthly screening. Limitations of the study include the weak validation of the HSCL screening tool and lack of validation of the MSPSS screening tool within the context of Nepal and among this patient group; a limited number of group support sessions and evaluation interviews undertaken; and untested inter-rater reliability between the two counsellors performing screening at each centre. Finally, we only evaluated fidelity to the intervention using counsellor diaries which has limited validity.

Our findings suggest we implemented an overly complicated screening process. We provided HAP to only a small proportion of patients (12/135). Use of a single screening tool might have increased the number of patients identified for HAP, because using a two-stage screening process (HSCL/MSPSS and then PHQ-9) prior to undertaking HAP led to conflicting indications from screening tools and a lack of clarity about which patients were eligible for counselling. Because of this some patients who may have benefitted from counselling did not receive it. We therefore recommend a single screening process using PHQ-9, because PHQ-9 has been well-validated in Nepal[22] as opposed to the weaker validation for HSCL there; and PHQ-9 screens for depression rather than anxiety, which aligns with HAP counselling which is designed as an intervention for depression and not anxiety. That said, the TB health workers did find the ultra-brief screening tool (Nepali version of PHQ-2) feasible in the current system. This could be considered as part of a screening process: the health workers screen using the PHQ-2 then refer screen positive patients to dedicated counsellors who uses the PHQ-9 to determine if a patient is eligible to receive a counselling intervention.

There are very few published studies reporting the feasibility of psychosocial interventions in MDR-TB patients so only limited comparisons can be made between our study and other evidence. Our finding that HAP is acceptable in MDR-TB patients in Nepal is in line with the findings of the previous HAP study conducted for patients with probable depression in primary care settings in India[16]. The requirement that patients attend the MDR-TB clinic regularly for MDR-TB treatment monitoring aligns with the HAP mode of delivery within health facilities. Unlike in the Indian RCT[17], we did not attempt to evaluate the effectiveness of HAP; patients in our study who received HAP did have improved PHQ-9 scores afterwards, but small numbers limit the conclusions that can be drawn. A study reporting on group counselling in Peru[23] found similar challenges to our study in recruiting and retaining patients in groups. Other similar findings include the fact that patients found it comforting to hear that other patients were experiencing similar problems and the beneficial use of cured patients in groups.

Our findings suggest that certain elements of the intervention package (information materials) may be beneficial for the psychosocial wellbeing of all the patients with MDR-TB and is feasible to be delivered by health workers in existing MDR-TB services in Nepal without additional resources. The other aspects of the intervention package (screening, group support and counselling) have some resource implications for Nepal’s NTP. This may be considered by the NTP given that the provision of psychosocial counselling and training of health workers in MDR-TB centres has been incorporated in the forthcoming strategy[10].

Provision of a dedicated counsellor in the MDR-TB centre, familiarity of the counsellor with the local language, private space to deliver counselling, motivation and support of the MDR-TB health workers for them to facilitate the intervention implementation and the need to have a single screening tool were some of the major factors that influenced implementation of the intervention package. Therefore, these factors must be taken into consideration before implementing at scale. Furthermore, although we excluded patients with severe depression (PHQ-9 score >19) from being eligible for HAP, the trial in India[17] did not make this distinction and found HAP appropriate for these patients. Therefore, it is likely HAP can be provided to all MDR-TB patients with PHQ-9 scores over 10. There may be scope within the NTP to train MDR-TB staff to manage suicide risk and deliver pharmacological treatments for depression but assessment of this possibility was outside the scope of our study.

Our findings lay a strong foundation for future research to test the effectiveness and cost-effectiveness of a behavioural activation counselling approach in the management of MDR-TB in Nepal, for instance through a randomised controlled trial. Aspects of the psychosocial package require further development such as the use of ultra-brief screening tools (e.g. PHQ-2) and the content of group support, including the use of cured patients. However, this psychosocial support package including screening and counselling shows promise as an intervention for patients receiving treatment for MDR-TB who have co-morbid depression.

## Supporting information

S1 Appendix MDR- TB patient information leaflet. (PDF)

S2 Appendix Reflective diary template for counsellors. (DOCX)

S3 Appendix Topic guide for interviews with patients. (DOCX)
