# Supplementary material for: Implementation of a psychosocial support package for people receiving treatment for multidrug-resistant tuberculosis in Nepal: A feasibility and acceptability study
Source: PLoS One. 2018 Jul 26;13(7):e0201163. doi: 10.1371/journal.pone.0201163 (PMC6062069; doi:10.1371/journal.pone.0201163)
Supplement: S1 Appendix — (PDF) [file pone.0201163.s001.pdf]

९. औषधि खान सम्झिनको लागि बिरामीले के गर्नुपर्छ ?

- प्रत्येक दिन एउटै समयमा औषधि खाने गर्नुहोस् ।
- औषधि खान सम्झनको लागि अलार्म राख्नुहोस् ।

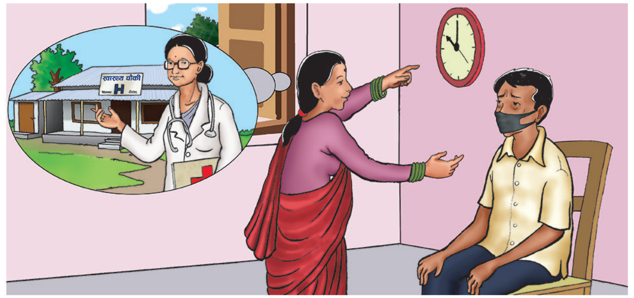

परिवारको कुनै सदस्य वा साथीलाई औषधि खान सम्झाउन आग्रह गर्नुहोस् ।

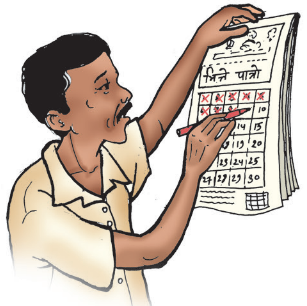

औषधि खाइसकेपछि क्यालेन्डरमा चिन्ह लगाउनुहोस्।

नोट : यदि औषधिको कुनै मात्रा छुटेमा एकै दिनमा दोब्बर मात्रा (dose) औषधि नखानुहोस् । साविक जस्तै औषधि लिइरहनुहोस् र छिटोभन्दा छिटो स्वास्थ्यकर्मीलाई यससम्बन्धि खबर गर्नुहोस् ।

एम. डि. आर. टि. बी. बिरामीमा पोषणको महत्व :

- स्वस्थ खाना खानुहोस् र पर्याप्त मात्रामा आराम गर्नुहोस् । दिनमा एकै पटकमा धेरै खानुभन्दा थोरैथोरै गरेर धेरै पटक खानुहोस् ।
- पखाला लागेमा बारम्बार बढी मात्रामा पानी र पुनर्जलीय झोल पिउनुहोस् ।
- मासु, र अण्डा मात्र भन्दा पनि स्थानिय स्तरमा सजिलै पाइने गेडागुडी, दाल, भटमासको सेवन गर्नुहोस् ।
- फलफुल र हरिया सागसब्जिहरु पनि लिने गर्नुहोस् ।
- सुई लगाएको अवधिभरि केरा र दही खानुहोस् ।

१३. एम. डि. आर. टि. बी. बिरामीले गर्नैपर्ने र गर्नहुने कुराहरू :

गर्नै पर्ने : • औषधि निश्चित समयमा नियमित खाने

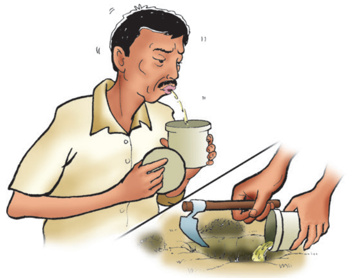

- खकार निश्चित ठाउँमा बिसर्जन गर्ने

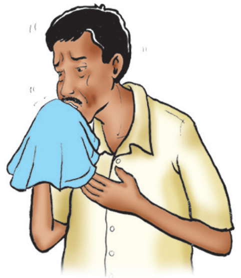

- खोक्दा वा हाच्छ्युँ गर्दा हरेक पल्ट मुख छोप्ने

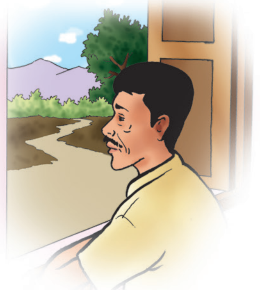

- हावा आवत-जावतको लागि झ्याल र ढोकाहरु खुला राख्ने

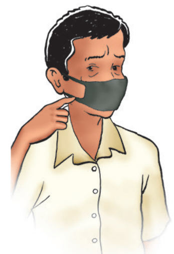

- मास्कको प्रयोग गर्ने

गर्नैनहुने :

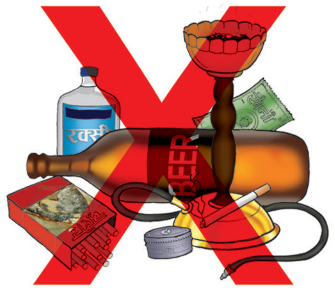

जाँडरक्सी, चुरोट र सुर्तीजन्य पदार्थ नखाने

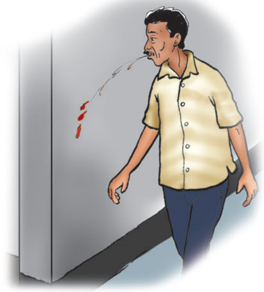

जहाँतहीँ नथुक्ने

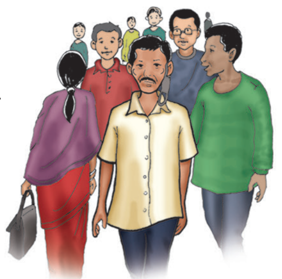

सकेसम्म अनावश्यक भीडभाडमा नजाने

याद राख्नुपर्ने कुराहरू :

- औषधि खाइसकेको केही महिनापछि बिरामीले सन्चो भएको महसुस गर्न थाल्छन् र औषधि बन्द गर्न सक्छन् । तर यो अवस्थामा टि. बी. का सबै जीवाणुहरु मरिसकेका हुँदैनन् । यसबेला औषधि खान छाड्दा अझ गम्भीर किसिमको क्षयरोग हुनसक्छ । त्यसैले स्वास्थ्यकर्मीले निको भएको नभनेसम्म औषधि खान नछोड्नुहोस् ।
- औषधि उपचारले राम्रो काम गरे नगरेको थाहा पाउन स्वास्थ्यकर्मीले भनेबमोजिम खकार जाँच गराउनुहोस् ।
- यदि कुनै अन्य रोगको औषधि खानुपर्ने भएमा क्षयरोगको औषधि खाइरहेको कुरा डाक्टरलाई बताउनुहोस् ।

एम. डि. आर. को उपचार स्वास्थ्य संस्थामा निः शुल्क छ ।

सेवा र सल्लाहको लागि नजिकको स्वास्थ्य संस्थामा सम्पर्क राख्नुहोस् ।

सम्पर्क :
HEALTH RESEARCH AND SOCIAL DEVELOPMENT FORUM
स्वास्थ्य अनुसन्धान तथा सामाजिक विकास मञ्च
HERD
थापाथली, काठमाडौं
फोन नं. : ०१-४२३८०४५, ०१-४१०२०७२

एम.डि.आर टि.बी. (MDR TB)

स्वास्थ्य जानकारी
एम.डि.आर. भएका व्यक्तिहरुका लागि

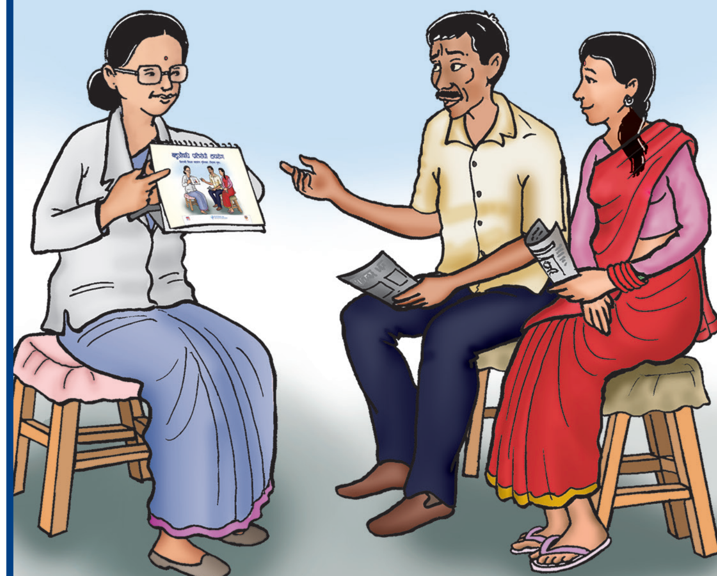

UKaid from the British people
HEALTH RESEARCH AND SOCIAL DEVELOPMENT FORUM
स्वास्थ्य अनुसन्धान तथा सामाजिक विकास मञ्च
HERD

१. टि. बी. भनेको के हो ?

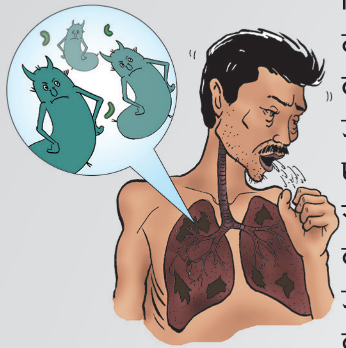

टि. बी. भनेको माईकोब्याक्टेरियम ट्युबरक्युलोसिस नाम गरेको जीवाणुबाट लाग्ने एउटा सरुवा रोग हो । यो रोग क्षयरोग भएका व्यक्तिले खोक्दा या हाच्छ्युँ गर्दा हावाको माध्यमबाट एक व्यक्तिबाट अर्कोमा सर्छ ।

२. एम. डि. आर. टी. बी भनेको के हो ?

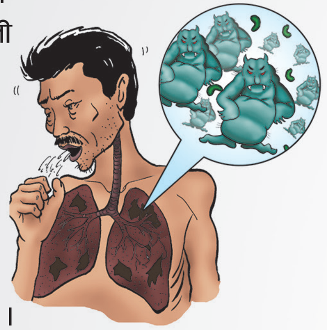

एम. डि. आर. टि. बी. भनेको टि. बी. का २ वटा शक्तिशाली औषधिहरु आइसोनियाजाइड र रिफाम्पिसिन (Isoniazid and Rifampicin) ले पनि मार्न नसक्ने जीवाणुद्वारा लाग्ने रोग हो । यसमा विरामीलाई टि. बी. को औषधिले निको पार्न सक्दैन ।

३. एम. डि. आर. टि. बी. कसरी हुन्छ ?

– स्वास्थ्यकर्मीले दिए अनुसारको टि. बी. को नियमित औषधि सेवन नगरेमा
– टि. बी. को उपचारको अवधि पूरा नगरेमा
– एम. डि. आर. टि. बी. भएका व्यक्तिले खोक्दा या हाच्छ्यु गर्दा सीधै हावाको माध्यमबाट

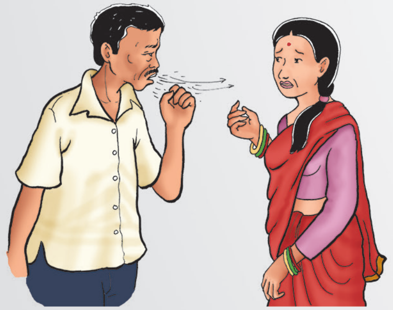

४. एम. डि. आर. टि. बी. का लक्षणहरु के के हुन् ?

साधारण टि.बी. को उपचार नियमित गर्दागर्दै पनि स्वास्थ्य अझै खस्कँदै जानु वा निम्न लक्षणहरु देखा परिरहनु ।

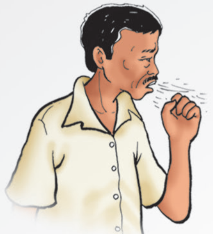

अ) खोकी लागिरहनु

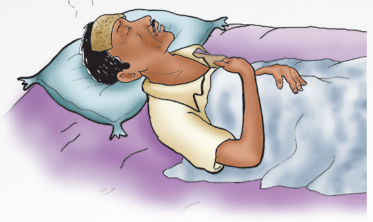

आ) राति पसिना आउनु / ज्वरो आउनु

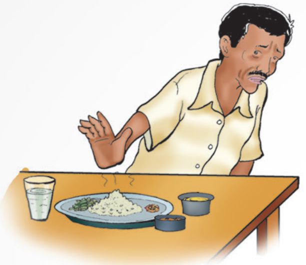

इ) खाना नरुच्नु

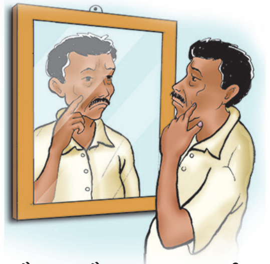

ई) तौल घट्दै जानु / थकाई लाग्नु

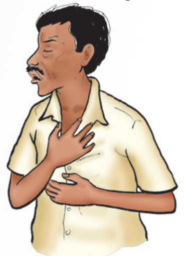

उ) छाती दुख्नु

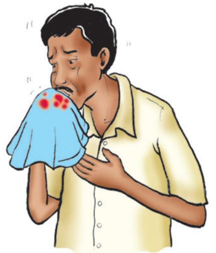

ऊ) खकारमा रगतको छिटा देखिनु

यदि तपाईले आफू या अरु कोही औषधि खाँदै गरेका साथीहरुमा यी लक्षणहरु भएको थाहा पाउनु भएमा तुरुन्त आफूले औषधि खाँदै गरेको स्वास्थ्य संस्थामा जानकारी गर्नुहोस् ।

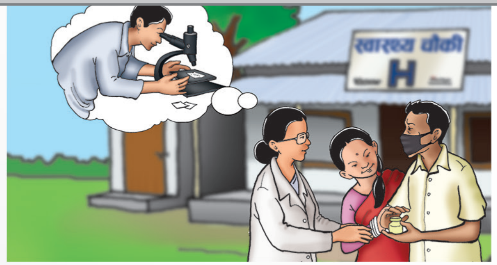

५. एम. डि. आर. टि. बी. को निदान कसरी हुन्छ ?

खकारको जाँच : अ) जिन एक्सपर्ट मेसिनबाट आ) कल्चर / डि. एस. टि. (Drug Susceptibility Test) बाट

६. एम. डि. आर. टि. बी. को बिरामीबाट कति समयसम्म अरुलाई यो रोग सर्ने सम्भावना रहन्छ ?

सामान्यतया औषधि खान शुरु गरेपछि अरुलाई सर्ने सम्भावना घट्दै जान्छ । जबसम्म खकार कल्चरको नतिजा नेगेटिभ आउँदैन त्यतिबेलासम्म यो रोग अरुलाई सर्ने सम्भावना रहन्छ । त्यो समय भनेको ४ देखि ६ महिना हुनसक्छ ।

- यदि तोकिए अनुसार नियमित औषधि खाएमा एम. डि. आर टि. बी. को उपचार गर्न सकिन्छ र यो निको हुनसक्छ ।
- एम. डि. आर टि. बी. को औषधि धेरै महंगो छ तर सरकारले नि:शुल्क वितरण गरेको छ ।

७. टि. बी. र एम. डि. आर. टि. बी. उपचारबीचको भिन्नता :

टि. बी.	एम. डि. आर. टि. बी.
• उपचार अवधि ६-८ महिना हुन्छ ।<br>• थोरै औषधिहरु खानु पर्ने हुन्छ ।<br>• औषधिका प्रतिकुल असरहरु कम हुन्छन् ।	• उपचार अवधि २०-२४ महिना हुन्छ ।<br>• धेरै औषधि र सुई लिनुपर्ने हुन्छ ।<br>• औषधिका साधारणदेखी गम्भीर खालका प्रतिकुल असरहरु हुन सक्छन् ।

• टि. बी. र एम. डि. आर. टि. बी. दुबैको उपचार प्रत्यक्ष निगरानीमा हुनैपर्छ ।
• यदि तोकिएका उपचार विधि अनुसार उपचार नभएमा वा नगरेमा रोग झन् गम्भीर हुन सक्छ ।

८. एम. डि. आर. टि. बी. औषधिहरुको प्रतिकुल असरहरु :

– तनाव/व्याकुलता
– मनमा अनावश्यक कुराहरु खेल्नु
– जोर्नीहरु दुख्ने
– छालामा विमिरा आउने / चिलाउने

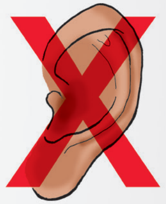

कान नसुन्ने

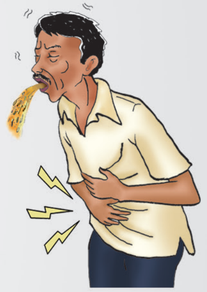

पेट दुख्ने र बान्ता हुने

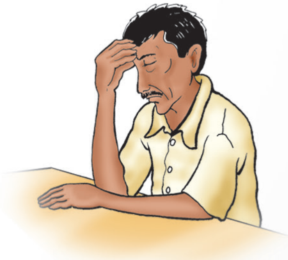

उदासीनता

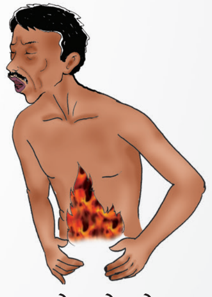

पेट पोल्ने

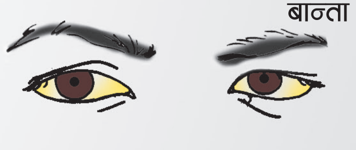

आँखा पहेंलो हुने

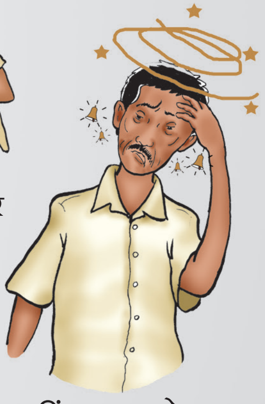

रिंगटा लाग्ने

नोट : यदि तपाईले यस्ता लक्षणहरु महसुस गर्नुभएमा स्वास्थ्य संस्थामा खबर गर्नुहोस् । स्वास्थ्यकर्मीको सल्लाहबिना औषधि खान नछोड्नुहोस् ।
