# Supplementary material for: Implementation of a psychosocial support package for people receiving treatment for multidrug-resistant tuberculosis in Nepal: A feasibility and acceptability study
Source: PLoS One. 2018 Jul 26;13(7):e0201163. doi: 10.1371/journal.pone.0201163 (PMC6062069; doi:10.1371/journal.pone.0201163)
Supplement: S2 Appendix — (DOCX) [file pone.0201163.s002.docx]

**Diary Format for MDR TB study**

Date:

**I) Venue:**

**II) Clinic hours:**

Opening time of the treatment centre

Closing time of the treatment centre

**III) Feasibility in terms of Patients/work load:**

1. Total number of MDR TB patients:
2. Number of staffs in the treatment centre:

**IV) Feasibility of the intervention:**

**Counselling using a flipbook:**

1. Clarity amongst the health workers about various processes of intervention
2. Number of patients receiving counselling using a flipbook:
3. Number of family members receiving counselling using the flipbook:
4. Any difficulty faced while using the flipbook
5. Any feedback/observation/comments on the mode of delivery of advice using the flipbook:

Time taken to counsel using the flipbook (average):

1. Number of patients receiving leaflet:
2. Number of family members receiving leaflet:
3. Any general comments on how the intervention appeared to be received by patients:

**Counselling with the counsellor:**

1. Number of patients receiving the HAP session from the counsellor:
2. Average time taken for the HAP session:
3. Waiting time before seeing the counsellor (average):
4. Any environmental or physical constraints
5. Average time taken for counselling
6. No. of patients who brought their family members
7. Personal difficulties felt as a counsellor

1. Any other significant observation (Personal feedback)
2. Any deviation from the planned intervention or any new adaptation conducted to make the intervention more feasible or practical? Anything that did not go according to plan?

Any general comments on how the intervention appeared to be received by patients:

Any lesson learnt for yourself?

Other interventions:

**Any other intervention given:**

Number of people who attended the intervention:

Patients

Family members

Topics covered

General remarks

1. Personal difficulties felt as a counsellor
2. Any other significant observation (Personal feedback)
3. Any deviation from the planned intervention or any new adaptation conducted to make the intervention more feasible or practical? Anything that did not go according to plan?

Any general comments on how the intervention appeared to be received by patients:

Any lesson learnt for yourself?
