# Supplementary material for: Implementation of a psychosocial support package for people receiving treatment for multidrug-resistant tuberculosis in Nepal: A feasibility and acceptability study
Source: PLoS One. 2018 Jul 26;13(7):e0201163. doi: 10.1371/journal.pone.0201163 (PMC6062069; doi:10.1371/journal.pone.0201163)
Supplement: S3 Appendix — (DOCX) [file pone.0201163.s003.docx]

**Psychosocial Interventions for people with MDR TB in Nepal: Interviews with patients**

**Topic Guide**

**Aims**

- To capture the experiences of people with MDR-TB receiving psychosocial interventions in the Nepal context to inform future developments of the intervention.

**Objectives**

- To identify the acceptability to patients of receiving psychosocial interventions
- To identify which aspects of the intervention helped and which didn’t.
- To identify how these interventions could be improved.

**Introduction to interview**

Describe the project being undertaken in psychosocial interventions in Nepal and the importance of learning what works and what does not work, so we can improve the intervention. Explain that we want to help make the service as good as possible and their views will help future patients.

*Ensure the person is happy to be interviewed and recorded. State the confidentiality of the interview.*

**Initial detail questions (Ice breakers)**

1. How was your MDR TB diagnosed and have you faced any difficulties during treatment?
2. What clinics did you attend for your TB care?
3. What support did the in-charge give you?
4. Did you have someone at the TB clinic asking you about your psychosocial (heart-mind) problems?
5. We have been conducting a research study. Were you told about this by anyone at the clinic or given any information about this study?

**Opening question – Psychosocial (heart-mind) Problems**

Have you experienced psychosocial problems ('heart-mind' problems – ‘manko samasya’) while you have had MDR TB? Please describe what these are?

Prompt: e.g. Heart-mind pain (‘man dukhne’), feeling sad (‘manmaa dukha laagchha’), ‘tension’, frustration (dikka) or despair (niraash).

Did any of these issues have any impact on your TB treatment course? If so, how?

**Receiving support from the TB service**

Do you feel that the TB service has supported you with these psychosocial problems? Do they seem to understand about these heart-mind problems? Did they help you with these heart-mind problems? How?

Did you receive any help (interventions) for heart-mind problems from Pradip/Sanjeev? What help?

Prompt: Did you get Education leaflet? Support group? Telephone support? HAP counselling?

How do you feel about the TB staff (counsellors) giving you counselling? Would you prefer it to be someone else (in charge)? Why?

**Evaluating the Screening process**

Were you regularly asked questions by Pradip/Sanjeev about your psychosocial (heart-mind) problems?

How did you find the questions you were asked regularly by Pradip/Sanjeev in the screening tools?

How did you feel when you were being asked these questions?

**Evaluating the Interventions**

If education materials, then –

- Did you receive (have you seen) a flipbook? How and when?
- What did you understand from the flipbook?
- Was it helpful? How did it help you?
- Do you have suggestions on how we can improve it?
- Did you receive an information leaflet? Who gave it to you and when?
- Was it helpful? How did it help you?
- Did you take it with you when you left the clinic and use it with your family?
- Do you have suggestions on how we can improve it?
- Did you get an information leaflet for your family members?

If the support group (group counselling), then –

- Did you have group counselling at the TB clinic? Who told you about it?
- What were you told before you received this group counselling?
- Did you want to do the group counselling?
- In what ways did you find the support group helpful?
- In what ways was the support group not so good? How could it be better?
- Do you think this should be offered to all TB patients and why? How frequently?
- Would you recommend the support group to other people with heart-mind problems? Why?

If the HAP counselling, then –

- Did you have the HAP/detailed counselling sessions? How many did you have?
- What were you told about the counselling before you started? What information?
- What was done during those sessions? Please describe. (Did you make goals and follow agreed actions with the counsellors?)
- In what ways did you find the counselling helpful?
- In what ways was the counselling not so good? How could it be better?
- Did you find the counselling sessions easy to understand and take part in?
- Did you do anything in between the counselling sessions related to what you had talked about in the session?
- Was the location of the counselling comfortable and suitable for you?
- Was the length of the session and their frequency suitable?
- Do you think the counselling sessions actually helped you with your psychosocial problems?
- Would you recommend the counselling to other people with heart-mind problems?

**Family involvement**

Did you come to the clinic with your family? Were they supportive with your TB treatment?

Did your family understand and help you with your heart-mind problems?

What can the TB service do that will help your family understand your TB treatment and the psychosocial (heart-mind) problems and to support you better?

**In the future, how can we best help other people with MDR-TB who have heart-mind problems?**

**Any other comments about these things?**

**Ending -** “Thank You for your time…”
